# Barriers encountered and implementation strategies deployed to facilitate real-world remote symptom monitoring in patients receiving active cancer treatment

**DOI:** 10.3389/frhs.2026.1875180

**Published:** 2026-08-12

**Authors:** Gabrielle B. Rocque, Nicole L. Henderson, D’Ambra N. Dent, Tanvi V. Padalkar, Emma Hendrix, Catherine S. Smith, J. Nicholas Odom, Tara Kaufmann, Chao-Hui Sylvia Huang, Chelsea McGowan, Jennifer Young Pierce, Ethan M. Basch, Justin D. Smith, Angela M. Stover

**Affiliations:** 1 Department of Medicine, Division of Hematology and Oncology, University of Alabama at Birmingham, Birmingham, AL, United States; 2 Department of Medicine, Division of Gerontology, Geriatrics and Palliative Care, University of Alabama at Birmingham, Birmingham, AL, United States; 3 O’Neal Comprehensive Cancer Center, Birmingham, AL, United States; 4 School of Nursing, University of Alabama at Birmingham, Birmingham, AL, United States; 5 Dell Medical School, University of Texas at Austin, Austin, TX, United States; 6 Mitchell Cancer Institute, University of South Alabama; Mobile, AL, United States; 7 Department of Health Policy and Management, University of North Carolina at Chapel Hill, Chapel Hill, NC, United States; 8 Department of Population Health Science, University of Utah, Salt Lake, UT, United States; 9 Lineberger Comprehensive Cancer Center, University of North Carolina at Chapel Hill, Chapel Hill, NC, United States

**Keywords:** CFIR, ePROs, implementation strategies, multi-level, mixed method, remote symptom monitoring, ERIC, Enhancing Oncology Model (EOM)

## Abstract

**Background:**

Remote symptom monitoring (RSM) with electronic patient-reported outcomes (ePROs) impacts patient and health system outcomes, yet data is lacking on real-world barriers to RSM and implementation strategies employed to address barriers.

**Methods:**

Guided by the updated Consolidated Framework for Implementation Research 2.0 (CFIR), this two-site implementation evaluation of early RSM implementation and scale-up aimed to evaluate barriers, facilitators, and implementation strategies. Patients and clinical team members were interviewed. Transcripts were coded in NVivo using directed content analysis to categorize barriers and facilitators. The Longitudinal Implementation Strategy Tracking System (LISTS) method was used to track implementation strategies deployed. Incidences where a barrier was reported were linked to strategies employed, creating key barrier-strategy combinations. Kappa for agreement between coders (all >.80).

**Results:**

40 patients and 30 clinical team members (physicians, nurses, nurse practitioners, non-clinical navigators, administrators) completed interviews. Interviewees identified 47 barriers in the CFIR domains. Eight key barrier-strategy combinations were identified by both patients and clinic teams; 13 additional combinations were identified by both groups. Amongst these 21 barrier-strategy combinations, primary barriers addressed related to the innovation (19%), outer setting (10%), the inner setting (19%), individuals (10%), and implementation processes (43%). Unaddressed barriers persisted despite use of multiple implementation strategies.

**Conclusion:**

Implementing RSM is complex and requires combinations of implementation strategies to overcome barriers at multiple CFIR levels. Future work is needed to leverage learnings from rigorous implementation studies to facilitate the RSM adoption.

**Trial registration:**

NCT04809740, registered 3/18/2021

## Introduction

Implementation of electronic patient-reported outcomes (ePROs), such as weekly remote symptom monitoring (RSM), is now required by the Center for Medicare and Medicaid Services (CMS) as a participation requirement for their value-based payment reform demonstration project, the Enhancing Oncology Model (EOM) (1). This requirement stems from multiple randomized clinical trials conducted in the United States (US) and internationally that support RSM to improve patient quality of care and patient outcomes. In the seminal trial by Basch and colleagues, RSM in patients undergoing active cancer treatment led to significantly improved symptom control, quality of life, length of time on treatment, hospitalization rates, and overall survival when compared to standard of care for patients with metastatic solid tumors (2, 3). In studies conducted in France, patients with Stage II-IV lung cancer completing RSM resulted in earlier detection of relapse and a 7.6 month survival benefit (4, 5). Further, a nurse-navigator intervention leveraging RSM resulted in improved patient experience, hospitalization rates, quality of life, and survival in patients receiving oral treatment for cancer (6). In Canada, Barbera and colleagues demonstrated that clinic-based ePRO symptom assessment could improve linkage to palliative care services, with reductions in emergency room visits and hospitalizations (7, 8). These smaller studies are complimented by the larger, cluster-randomized PRO-TECT trial which tested RSM across 52 US sites, finding improved symptom control and quality of life (9).

While these clinical trials demonstrate the effectiveness of RSM to improve care delivery, real-world experience on implementing RSM at scale for entire cancer center populations is lacking. This is a significant gap because at least 41 practices across the US are participating in the EOM and must fully implement ePROs and RSM into standard of care practice by 2026 and a second call for EOM entry is currently open. Texas Oncology provided the first large-scale experience with ePROs delivered outside the clinical trial context, enrolling >4,000 patients across their 210 practices with a 64% completion rate for weekly ePRO assessments (10). The University of Alabama at Birmingham (UAB) and University of South Alabama Mitchell Cancer Institute (MCI) have also implemented standard-of-care programs, sharing intervention adaptations that support integration into standard of care (11). The program demonstrated enrollment rates in diverse populations >90% of patients approached, survey completion rates of 80%, and >70% of alerts closed by nursing staff within the goal time for the institution (12, 13). These initial evaluations focused largely on adapting the evidence-based complex health intervention to the real-world context and reporting feasibility and early effectiveness results, rather than understanding the interplay between barriers, implementation strategies, and implementation outcomes within real-world settings.

In addition to adapting RSM to real-world settings, implementation strategies must be employed to overcome barriers and to tailor the intervention to local contexts and workflows to facilitate adoption, implementation, sustainability, and expansion of the program (14, 15). While guidance and frameworks for ePRO implementation based on expert opinion (16, 17) and broad compilations of implementation strategies are available (18), pragmatic data on implementation strategies gathered during real-world implementation efforts are lacking (19). This type of data is critically important given the complexity of RSM implementation and know challenges with workflow burden, alert fatigue, staffing limitations, system interoperability, and digital literacy challenges (11, 20, 21). In our previous work, we identified digital health literacy had lower comfort with all technology tasks than clinical teams (21). When implementing interventions in routine clinical care, multiple strategies are often leveraged simultaneously or sequentially to support implementation and use of these strategies may vary depending on specific clinical contexts (22). Given the need for cancer practices to meet requirements for payment reform and improve patient care, there is an urgent need to provide evidence-based guidance in RSM implementation by further characterizing barriers and the strategies used to address them. Hence, the objective of this study was to use the CFIR (23) to characterize real-world barriers, employed implementation strategies, and the implementation outcomes targeted to guide future launch and scale-up of standard-of-care RSM.

## Methods

### Overview

This interpretive, implementation-focused qualitative evaluation (24) examined (1) patient and healthcare team experiences with RSM, as well as perceptions of barriers and facilitators, and (2) implementation strategies utilized to target barriers identified. The study was conducted at UAB and MCI in Alabama, where 26% of patients are Black and 16% are living in poverty (25). UAB is the only National Cancer Institute-designated cancer center in the state. The program is described in full elsewhere (26), but in brief, patients are enrolled by non-clinical navigators employed by the health system in an RSM program. Patients then complete weekly, brief symptom assessments sent by text or email. Moderate to severe or worsening symptoms directly alert clinic nurses within their natural EMR workflow. Nurses subsequently respond to symptoms using institutional standard protocols and procedures. The ePROs and alert mechanism was integrated into the electronic medical record to facilitate implementation such that providers could access the ePROs within the patient's chart. This standard of care implementation was initiated in 2021, and 1,758 patients were enrolled across the two institutions by the end of 2023.

Two data sources were leveraged for this analysis of RSM implementation: (1) semi-structured interviews with patient and healthcare team participants (Supplementary Appendix S1) and (2) documentation of implementation strategies employed by the clinical team. The study, including informed consent, was approved by the University of Alabama at Birmingham Institutional Review Board (IRB-300007406) in accordance with the Declaration of Helsinki and Good Clinical Practice guidelines (GCP) registered on clinicaltrials.gov (NCT04809740, 3/18/2021).

### Qualitative interviews

Barriers, facilitators, and perspectives on implementation strategies were assessed during 30–60-minute semi-structured interviews that were conducted in person, over the phone, or on zoom. Interviews occurred at 2 time points (Year 1 and Year 2), and participants were not repeated across time points. The goal sample size was 20 patients and 10–15 clinical team members per year. Patients were identified by members of the clinical and research teams through screening of clinic lists. Patients were approached during routine clinic visits by a research coordinator and were purposively sampled to represent a broad range of patient backgrounds in terms of race, education level, and socioeconomic status. Clinical team members were identified by the study and site PI (GR, JYP) to represent a variety of clinical roles (navigators, nurses, physicians, administration) and were invited to participate by email. Informed consent was obtained for all participants. Patients were compensated $50 for their time; clinical team members were not compensated.

A semi-structured interview guide, based on the CFIR (23) and Powell's compilation of implementation strategies, focused on experiences participating in the RSM implementation was developed for patients and clinicians. This guide was based on existing evidence and consultation with clinical and implementation experts but was not pre-tested with patients or clinicians. All interviewers had prior qualitative training and experience conducting interviews and were trained on the interview guide. Year 1 patient interviews (*n* = 20) were conducted by a clinical psychologist (SH) and clinician interviews (*n* = 15) were conducted by the site PIs (GR; JYP), each interviewing at the alternate site to minimize potential for bias related to personal relationship with the interviewers. Interviews were recorded, transcribed to blind coder to participant identity, and inductively content-coded by 2 independent coders (NL; EH, research assistant) using a constant comparative method in NVivo software (27). The open-coding schema was reviewed with the principal investigator (GR) to formalize a codebook, then another round of focused coding occurred.

In Year 2, the interview guide was expanded to build upon themes identified in year 1 interviews and to incorporate questions specifically related to employed implementation strategies. Patient interviews (*n* = 20) were conducted by a medical anthropologist (NH), and clinician interviews (*n* = 15) were conducted by an oncologist external to the participating institution (TK) who was engaged to maximize clinician comfort and likelihood to disclose barriers. Three independent research assistants (EH; TP; CS) utilized a combination of the year 1 codebook and open coding to identify emergent themes in the maintenance phase of implementation. The final codebook was reviewed in conjunction with the research team (GR, NH), and a final round of focused coding was performed on year 1 and year 2 interviews to ensure consistency. The interrater reliability was assessed using the Kappa statistic calculated in NVivo for each set of interviews.

### Implementation strategy capture

Implementation strategies were captured using the Longitudinal Implementation Strategy Tracking System (LISTS) developed by Smith and colleagues (22). This method enables the capture and reporting of implementation strategies from the Expert Recommendations for Implementing Change (ERIC) compilation (18) according to actor, action, action target, temporality, dose, and barriers addressed using Proctor's strategy reporting guidance (27). It further allows the linkage of implementation strategies to the barriers they address utilizing the CFIR (15, 23) and implementation outcomes they target according to the Implementation Outcomes Framework (28). Two coders (GR; DD) utilized field notes from meetings, activity logs, and meeting transcripts to retrospectively characterize strategies and addressed barriers in years 1 and 2 of RSM implementation. GR is an oncologist and health services researcher with formal training in implementation science and public health, while DD has an MBA and functioned as the primary RSM trainer. Two additional coders from the operational team reviewed for accuracy and completion (SI, program manager; CM, research assistant). Summary categories and operational definitions were reviewed by PhD implementation experts external to the participating sites to ensure accurate capture of terminology (AS, BW, DH). After the initial input, codes were reviewed quarterly to ensure continued accuracy.

### Integration

The qualitative codes were then reviewed in parallel with the tracked implementation strategies, linking incidences where the qualitative data explicitly discussed either the barrier addressed by the implementation strategy or where the participant discussed the implementation strategy itself. Incidences where a barrier was reported were linked to strategies employed, creating key barrier-strategy combinations. This triangulation of data from the investigative team (implementation strategies) with the patients and clinical team members (interviews) allowed for richer contextualization and better understanding of the impact of experienced barriers and employed implementation strategies. Disagreements were managed through consensus discussion with the entire multidisciplinary team. Audits of decisions were kept through a memo tracking process.

## Results

### Patient and clinical team perspectives

A total of 40 patients and 30 clinical team members participating in RSM at UAB and MCI were interviewed between December 2021 and February 2024 (Table 1). The patients were balanced across both sites and were between 24 and 77 years old. Consistent with the demographics of Alabama, 27.5% of patients were Black/African American (AA). Due to initial implementation in breast (UAB) and gynecologic cancer clinics (MCI) in Year 1, participants were predominately female (88%). The clinical teams interviewed included physicians (30%), nurses (27%), nurse practitioners (7%), non-clinical navigators (23%), and administrators (13%). Among the clinician participants, most were female (73%) and White (77%). There was good agreement between reviewers (kappa for clinicians 0.87 and 0.98; kappa for patients 0.86 and 0.80 in years 1 and 2, respectively).

**Table 1:** Participant demographics.

Variable	Patients Total sample: *N* (%)	Clinical teams Total sample: *N* (%)
Sample Size	40	30
Age: Under 30	1 (3%)	5 (17%)
30–45	12 (30%)	18 (60%)
46–65	14 (35%)	4 (13%)
Over 65	13 (32%)	0 (0%)
Declined to answer		3 (10%)
Sex: Female	35 (88%)	22 (73%)
Male	5 (12%)	8 (27%)
Race: White	29 (72%)	23 (77%)
Black	11 (28%)	6 (20%)
Prefer Not to Answer	0 (0%)	1 (3%)

Across both patient and clinician samples, 47 barrier themes were identified in the CFIR (23) domains of the innovation (43%), outer setting (4%), inner setting (19%), individuals (6%), and implementation process (28%). These barriers included 21 key barrier-strategy combinations where participants identified how implementation strategies were deployed to address the barrier (Tables 2a,b,c,d). There were 26 unaddressed barriers without an associated implementation strategy (Supplementary Table S1). Eight key barrier-strategy combinations were discussed by both patients and clinicians.

**Table 2A:** Barriers and implementation strategies related to inner setting, implementer characteristics, and delivery setting processes (planning).

Specific Barrier	Barrier Quote	Action (Strategy)	Action Quotes
Slow Technology* (Inner Setting/ Structural)	"Yeah, I definitely think it's important, and I think that unfortunately, ours at MCI has been slow.” MCI Provider 6	**Upgraded technology** (Change Physical Structure and Equipment)	"We had some issues with it connecting to the internet and not being able to respond. That was a while ago though, and we haven't had those issues since, so it's been good since [we upgraded.” MCI Provider 14
**Technology Feels Impersonal** (Implementer Characteristics: Knowledge & Beliefs)	"If I had a very severe side effect or symptom, I think I would want to call to speak with a person to discuss it rather than typing some words.. I think that it's just a learning thing and we are very comfortable with phone calls and it feels very personable with the phone call.” MCI Providers 3 and 4	**Nurse responds to Alert via Call** (Revise Professional Roles)	"I'm really, I am so grateful for that Wednesday contact. I really am. I picked Wednesday, and I changed it, I changed the day, because I figured out my bad day and I wanted to be contacted on my bad day.” UAB Patient 19
Provider Forgetfulness* (Implementer Characteristics: Other)	"Because we're busy. Sometimes we forget. Sometimes our navigators will forget. Sometimes our nurses will forget.” UAB Provider 6	**Nurse champions send email reminders** (Remind Clinicians)	"She's [the nurse champion] the one that if people forget to close alerts, she's sending out reminders.” UAB Provider 13
Not Paying Attention during Training* (Planning)	"They're so busy that it's like they're just trying to get through it honestly. “ MCI Provider 6 “It's not that they were not educated. That information was buried in probably a four-hour-long video where you were half asleep.” UAB Provider 15	**Hands-on, in-clinic training** (Make Training Dynamic); **Trainer available for real-time questions** (Provide Ongoing Consultation); **Develop Videos and Cheat sheets** (Develop and Distribute Educational Materials)	"I will set a time aside to go down and meet with them in clinic, and I'll pull up patients who have unclosed alerts and we will go through and I will go over how they need to respond, whether they've opted for a callback or not.” MCI Provider 13 “I've actually seen a more positive turnaround with the cheat sheets and having a resource to come to to say, “Hey, I don't understand this,” or, “How do I know if this patient qualifies or not?"“ UAB Provider 3

**Table 2B:** Barriers and implementation strategies related to delivery setting processes (engaging).

Specific Barrier	Barrier Quote	Action (Strategy)	Action Quotes
**Access to Smart Phone/Data** (Engaging)	"There are many patients, and especially older patients that are on Consumer Cellular with Target and they don't have a smart device.” MCI Provider 5 "At first they asked me if I had a smartphone and I didn't have one of those, just a flip phone.” MCI Patient 6	**Apply for BCBS funds to provide smart phones** (Access New Funding; Change Physical Equipment)	"So, if I contact that patient and they say, “Well, hey, I don't know how I'm going to do my surveys because I don't have a phone.” And I would tell them that that's no problem because we are able to get you connected with the phone and to show you how to use it.” UAB Provider 9
Buy-In Low Among Providers* (Engaging)	"Honestly, when I was hired, I was trained by a nurse who was not very thrilled about PROmpt. She made it sound like it was going to be awful and I was going to hate it, so she kind of made it a negative experience.” UAB Provider 3	**Integrated lead nurses on each site onto project team** (Identify and Prepare Champions)	“Oh, for sure. We have two really great [champions]. They are both so just supportive, accommodating. One sometimes will be like, “Hey, I have this new patient starting treatment, she's going to be really good for participating in remote symptom monitoring,” and stuff like that.” MCI Provider 13
**Digital Literacy** (Engaging)	"Because like I said, I wasn't very good on the computer.” MCI Patient 6	**Engage caregivers in survey completion** (Involve Family Members)	"I get my daughter to do most the technical stuff on the phone and stuff, because she's younger and she knows all this technical stuff.” MCI Patient 8
**Introductory Materials Not Used/Useful** (Engaging)	"I don't think I have any pamphlets. I do have just the step-by-step instructions on how to access their account, what they're using.” MCI Provider 5 "I don't believe I got that.” UAB Patient 9	**Videos, magnets, and additional introductory material for patients** (Develop and Distribute Educational Materials)	"Oh, I got it hanging up on my fridge, so I definitely use them.” MCI Patient 13
**Patient Physical and Psychological State during Approach** (Engaging)	"They don't know what to expect. And so I do know that sometimes surveys can be overwhelming for people.” UAB Provider 3 "It was my first treatment and I was extremely nervous. I remember I was messing up trying to put it in my phone, and I had to give her my phone to let her set everything up because I was just a nervous wreck.” MCI Patient 9	**Approach in clinic rather than in infusion and addition of online self-enrollment option** (Change Service Site)	"She came in to talk, I had an appointment set up with my nurse practitioner to talk about what to expect from chemo. And then this person swooped in at the end of that appointment to talk to me about this. And she was super nice and had a packet of information and explained to me what it was and asked if I wanted to do it. And I was like, “Sure thing."“ MCI Patient 18
Provider Perception of Low Patient Buy-In* (Engaging)	"It takes both the patients and us to make this work, so we need our patients to be on board with it from the start.” UAB Provider 3 "I think getting patient buy-in to some degree. Some patients are all about it. Some are like, “I don't want to do another survey."“ UAB Provider 10	**Interviews with patient participants** (Obtain and Use Patient Feedback); **Presentation of patient feedback at Grand Rounds** (Conduct Educational Meetings)	"I also heard a presentation on it that [the physician champion] did, and learning that patients are more likely to say what their symptoms are and say what's going on via this survey than calling or sending a patient portal message. Made me think about it in a much more positive light.” UAB Provider 13
Unawareness or Dislike of New Program Features* (Engaging)	"Yeah, I was not aware of a snooze feature.” MCI Provider 8 "I don't want to snooze it and then something happens and it comes back on me.” MCI Provider 7	**Implementation team visits individual clinic to reinforce programmatic features** (Conduct Educational Outreach Visits); **Provide refresher training** (Conduct Ongoing Training)	“We learned about the silencer where you can mute, which I feel like for those patients that are having these chronic issues that are not severe, but we know that it's going on that don't want to call back, is a good way to lessen the load on the nurse. We learned about that.” UAB Provider 13

**Table 2C:** Barriers and implementation strategies related to delivery setting processes (executing).

Specific Barrier	Barrier Quote	Action (Strategy)	Action Quotes
Disrupts Workflow*	"It did mess with the flow for everybody” MCI Provider 9 “They would rather it come to the results, which I could understand that because you're so used to what you're used to.” UAB Provider 4	**Movement of Alerts from Messages to Results** (Facilitate relay of clinical data to providers)	"No, I think we've made some really good modifications, and I haven't heard any complaints recently.” UAB Provider 11
**Increased Workload/ Time Consuming for Patients**	"Every time I go for treatment, they give me a tablet that's got a boatload of questions on it, and I have to fill that out every time I go for treatment. That takes a minute, and it's like the other questions, they're all pretty much repetitive.” MCI Patient 9 “I just didn't want to be tied down on Tuesdays to doing this.” UAB Patient 16	**NCN led training on system and participation** (Prepare patients to be Active Participants)	"So going through that baseline survey with them, making sure that they understand that moderate and severe alerts automatically opt them into a callback. Show them how to opt out. Let them know to still complete your survey, even if you aren't having symptoms, so they can see that you don't have symptoms this week.” MCI Provider 13
Increased Workload/ Time Consuming for Providers*	"I think you hear people groan, and we all know plenty of [providers], who say, ‘it's just one more thing for me to do.'“ UAB Provider 8	**No Friday surveys** (Promote Adaptability of the Intervention)	"Make sure patients don't fill out surveys on Fridays.” UAB Provider 6
Need for Team Role Clarification*	"No, I think our confusion probably is with who was enrolling the patients.” UAB Provider 11	**NCN responsible for enrollment and survey capture** (Revision of Professional Roles)	"She's (NCN) in charge of our GI team and she has been the one kind of responsible for enrolling patients and making sure they understand how the program works.” UAB Provider 14
**Patient Forgetfulness**	"I mean, sometimes we call people and they're like, “Oh, I just forgot. I'll do it right now. But some of the people, they're like, “Oh, yeah, I'll do it. I'll do it later,” and then they don't.” MCI Provider 6 “But sometimes, I don't, and then forget about it, until it's too late at the end of the day.” MCI Patient 10	**Follow-up; Text/Email Reminders; In clinic re-approach by navigators** (Intervene to enhance uptake and adherence)	“And then they had to call me a couple days later and they were like, “Hey, were you going to sign up for this?” And I was like, “Oh, yeah.” So, there is probably that part, the follow-up was important.” MCI Patient 11
Slow Addressing of Alerts*	"A lot of my people, some of them are habitually late, you'll look in there and they've got symptoms that are open for weeks.” UAB Provider 2	**Nursing leadership required alert closure** (Mandated Change) **Provision of Monthly and Quarterly Alert Response Reports** (Use Audit and Feedback)	"When I talked to [the physician champion] for the training aspect of it, the most critical was, “We need to have these alerts taken care of within 48 h.” UAB Provider 3 “Yeah, we send [monthly and quarterly monitoring reports] out to our nurses monthly when we get them so they can kind of see where they're at.” MCI Provider 11

**Table 2D:** Barriers and implementation strategies related to intervention characteristics.

Specific Barrier	Barrier Quote	Action (Strategy)	Action Quotes
**Log-in Issues (Complexity)**	"I tried to log in, to do that, and couldn't log in and I couldn't remember what my login was.” MCI Patient 10	**ePRO vendor technical support; NCNs and Local Tech Support** (Provide Technical Assistance)	"That kind of technical support, and very handy, very knowledgeable and using a very short time to get this resolved” MCI Patient 9
Need Ability to Document Persistent Symptoms* (Design Quality and Packaging)	"It has to do with if a symptom persists, not a new symptom. And there's no way to tell the symptom, as far as I recall, that there's no way to tell if a symptom that's been there every week, like insomnia, really hasn't gotten better or worse. There's no way to document that something is been there and it's not new.” UAB, Provider 5	**Removal of inactionable symptoms; addition of “snooze” button** (Promote Adaptability)	"Most of ours have fatigue and are always going to have fatigue. That's not, fatigue is something they deal with, so I'm glad they got rid of it. It's the other symptoms that go along with the fatigue that I worry about.” MCI Providers 11 and 12
Need Pause Button for Surveys* (Design Quality and Packaging)	"The only thing that I think I've mentioned is maybe where they can report that they're reporting from in the hospital, versus like from home.” UAB, Provider 3	**Alert is flagged if patient in hospital** (Promote Adaptability)	"I know they have the hospitalized feature, which I think is great for when a nurse sees that a patient is hospitalized” MCI Provider 13
Time Frame of Survey Questions* (Design Quality and Packaging)	"It's difficult because it asks about 7 days worth of symptoms when in fact those symptoms may have improved 5 days ago.” UAB, Provider 6	**Addition of “No Call Back” Question** (Promote Adaptability)	"So really working with the snooze and the callback feature, like those have been really game changers for us in reducing the workload burden for our staff.” UAB Provider 13

The types of barriers identified were related to the complexity of the intervention (I. Innovation), the broader context into which the intervention is implemented (II. Outer Setting), the specific context into which the intervention is implemented (III. Inner Setting), and their engagement in and execution in RSM (V. Implementation Process). Both groups emphasized the importance of how they were approached for enrollment, as well as barriers related to medical mistrust that persist in the Alabamian and Black-American context. In contrast, the clinical teams additionally emphasized key barrier-strategy combinations related to innovative aspects of the intervention design (I. Innovation), the intervention context (Inner Setting), the individuals involved (IV. Individuals), and the process of implementation (V. Implementation Process). They highlighted a desire to continue with how symptoms had always been managed without having to add and remember additional daily responsibilities, which was exacerbated by some who shared initially skeptical beliefs about the intervention impact. They also emphasized a multitude of inner setting barriers related to how the RSM-related technology was either not used or difficult to use, resulting in communication-related barriers. An additional 13 barrier-strategy combinations were identified by clinicians only, which also included barriers in the inner setting related to slowness of the technology within the electronic medical record system, which was addressed with software upgrades. There were no key barrier-strategy combinations that were exclusively discussed by patients. Examples from interviews of the barriers not addressed by implementation strategies are highlighted in Supplementary Table S1.

#### Implementation strategies

Implementation strategies were analyzed to understand how barriers were addressed during the scale-up process. From May 2020 until October 2023, 42 strategies were utilized (28 discussed in qualitative interviews and 14 not discussed). Supplementary Table S2 describes all implementation strategies, primary barriers with sub-barriers, and primary target. Of all strategies, 52% (22/42) were continued from time of early implementation until the end of the scale-up period. Discontinued strategies were predominately one-time or time-limited. Strategies discontinued intended to be time limited, included “change physical structure and equipment” related to acquiring tables, phones, and equipment to support the non-clinical navigators engaging with patients and “assess readiness and identify barriers and facilitators”, in which pre-implementation interviews were conducted with key clinical partners prior to launch as part of standard of care. Some continued strategies were modified in terms of cadence and format. For example, the strategy “conduct educational outreach visits” included a variety of sessions to target key clinical partners. Sessions included presenting at faculty meetings, nursing huddles, and individualized meetings with cancer working groups (e.g., breast cancer clinical team). The strategy was continued, but the timing and content evolved to meet the needs of the team, including both reinforcing for exiting clinicians and educating new clinicians as a part of structured onboarding to clinical practice.

**Figure 1:**
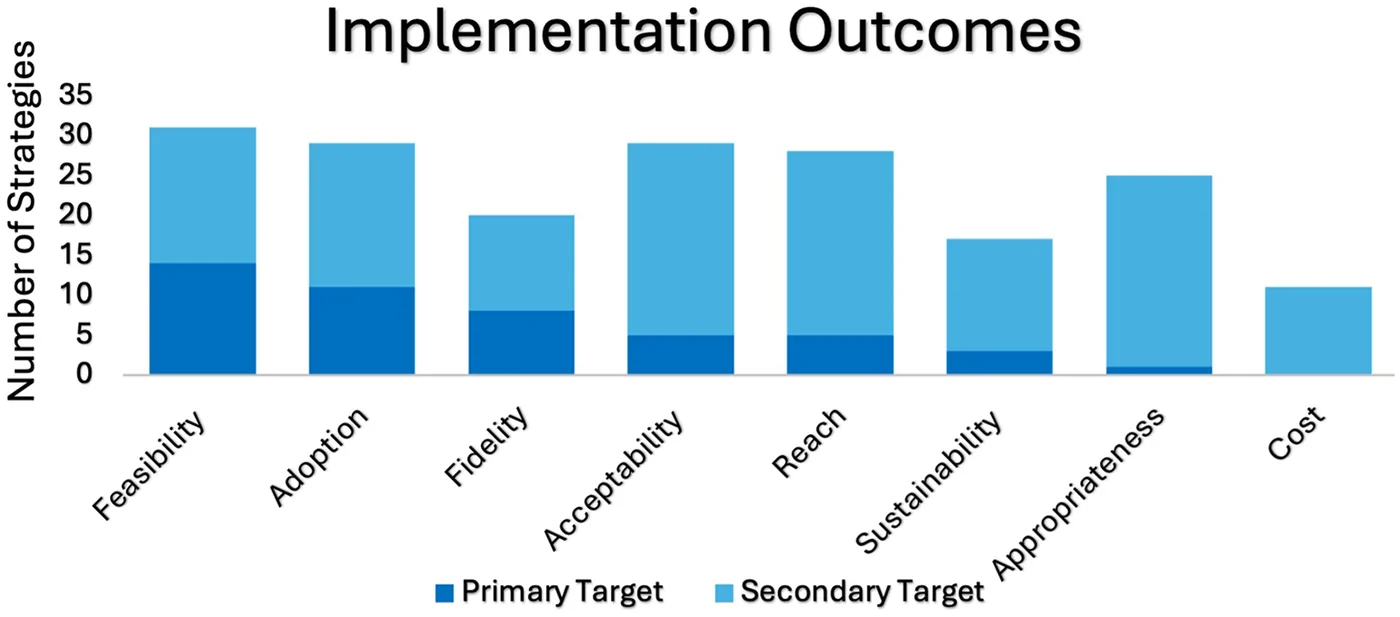
Implementation outcomes targeted by strategies used during remote symptom monitoring implementation and scale-up.

Overall, the most common implementation strategy category during this period was “develop stakeholder interrelationships”, followed by “train and educate stakeholders” and “use evaluative and iterative strategies” (Figure 1). Although these categories had the largest number of strategies, the implementation team used only approximately half of available strategies within these categories. The overarching category of “develop stakeholder interrelationships” included 47% (8/17) of potential implementation strategies: capture and share local knowledge, obtain formal commitments, promote networking, use advisory boards and workgroups, develop academic partnerships, inform local opinion leaders, identify and prepare champions, and use an implementation advisor. For the category of “train and education”, 54% (6/11) of potential implementation strategies were included: conduct educational outreach visits, develop educational materials, create a learning collaborative, distribute educational materials, make training dynamic, and provide ongoing consultation. The Iterative Strategy category emphasized development and deployment of quality monitoring tools to both address barriers and provide ongoing feedback, utilizing 54% (6/11) of strategies including: assess for readiness and identify barriers and facilitators, audit and provide feedback, develop and implement tools for quality monitoring, obtain and use patients/consumers and family feedback, and purposefully reexamine the implementation.

**Figure 2:**
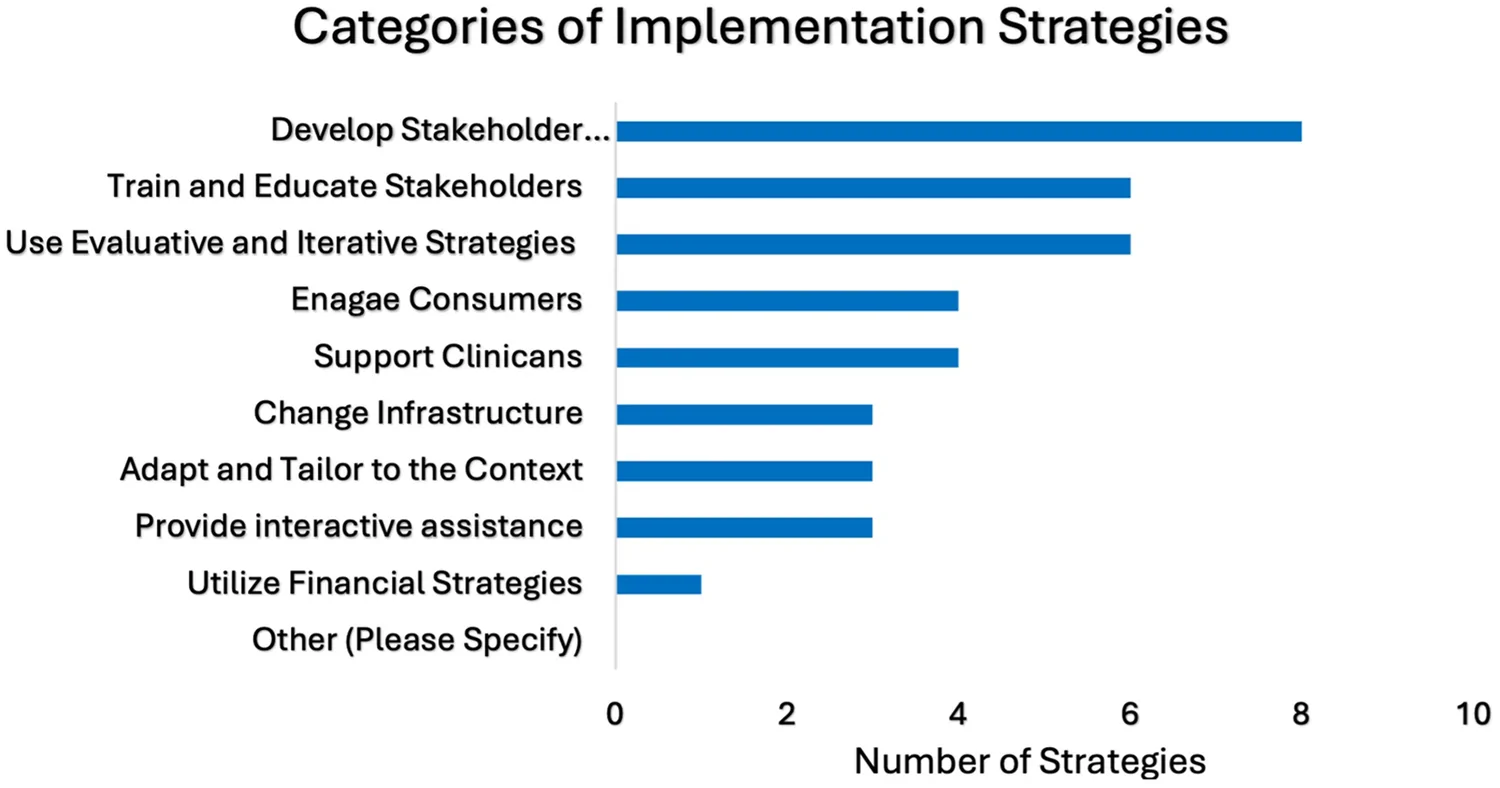
Categories of implementation strategies deployed during remote symptom monitoring implementation and scale-up.

This early implementation and scale-up phase primarily addressed 21 barriers common at program onset, with the primary barriers addressed related to the innovation (19%), outer setting (10%), the inner setting (19%), individuals (10%), and implementation processes (43%). This corresponded with the primary targets of the implementation strategies deployed, with feasibility (30%) and adoption (23%) as the most common targets. Other important outcomes related to longer-term use of the intervention, such as fidelity (17%), acceptability (11%), reach (11%), sustainability (6%), and appropriateness (2%), were less prominent targets. In contrast, the secondary targets were more balanced across targets towards a longer-term view of implementation outcomes that would be important for programmatic expansions and sustainability (Figure 2). Cost was not the primary target of any implementation strategy and was a secondary target in only 11 strategies. In several cases, multiple implementation strategies were used to address individual barriers.

**Figure 3:**
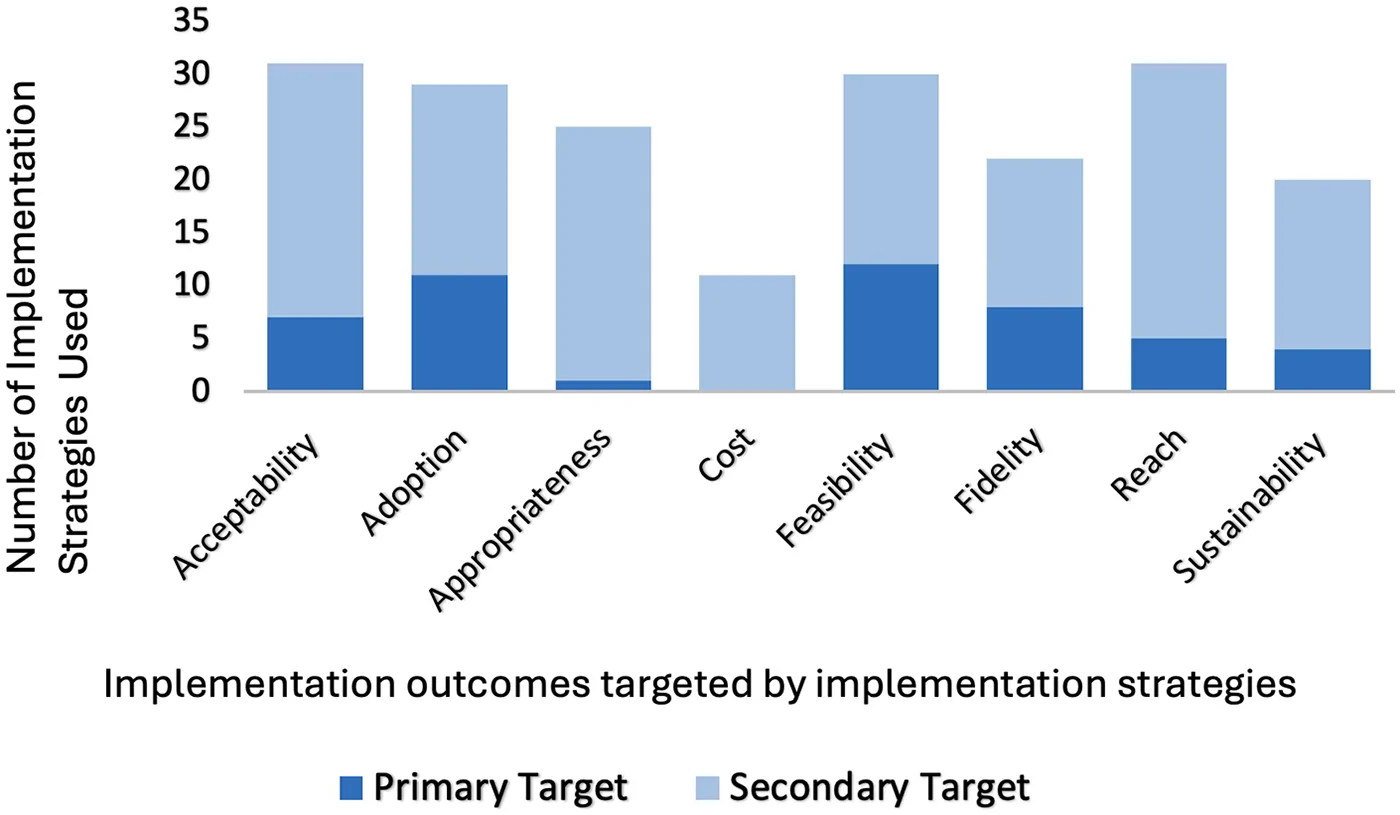
Primary and secondary implementation outcomes targeted by employed implementation strategies.

## Discussion

This study highlights the complex nature of implementing RSM as a health system intervention in a real-world setting, where barriers exist at multiple implementation levels. To address these barriers, over 40 discrete implementation strategies were deployed to target early implementation challenges within the initial 2 years. This high number of strategies is consistent with previous literature showing that numerous strategies are used to deploy other evidence-based interventions, such as stroke rehabilitation (29), suicide prevention (30), opioid risk management initiatives (31), and ePRO implementation (4). In addition to the finding that multiple implementation strategies are used, this study also identified that implementation strategies can be employed simultaneously or at various time points during implementation, highlighting the need for systematic capture of implementation strategies within program implementation. This also supports prior multi-site work finding implementation strategies evolved throughout RSM implementation (22).

The complexity of RSM underscores several key realities. First, determination of the numerical impact of an individual strategy in this context on traditional implementation outcomes (e.g., adoption of the intervention) is unlikely to be fruitful. However, role and relative contribution is ascertainable using qualitative research (32) and thorough documentation of target barriers and target implementation outcomes from the implementation team (22). Second, studies that test specific implementation strategies should concurrently consider capture of additional implementation strategies deployed, potentially using the LISTS method (22), to consider the interplay between strategies. This is particularly important given the finding from this study that barriers and implementation strategies were often not a 1:1 match, with some strategies being used to address multiple barriers and some barriers requiring multiple strategies. This real-world experience is consistent with prior research where Waltz and colleagues found that experts in the field vary substantially in their opinions of what implementation strategy best addressed individual barriers (33). Finally, individual participants (e.g., different patients) and subgroups (patients vs. clinicians) may vary in perceived strategy utility. For example, the in-person approach for ePRO participation was highly valued by some patients, whereas others preferred the web-based enrollment. At the same time, clinicians may vary in whether they appreciate the in-person approach based on whether they have the time for the navigator to meet with the patient either before or after the visit without disrupting workflow, as exemplified by the quote in Tables 2a, b. Ultimately, implementation strategy utility should be considered from the different perspectives of the agents involved in the intervention.

It is important to note that despite the many implementation strategies deployed, barriers remained unaddressed due to complexity, limited resources, or the early nature of this evaluation (early implementation and scaling). Furthermore, some implementation strategies deployed resulted in new barriers that warranted additional mediation. For example, the use of the “snooze” button (promote adaptability) to address alert fatigue was considered a highly successful strategy (“game changer”). However, suboptimal use of this tool where some nurses didn't use the feature became a new barrier, requiring a strategy of ongoing consultation to retrain and reiterate this option. Therefore, in real world settings, implementers should recognize that change is iterative, and a single assessment timepoint of barriers and implementation strategies is unlikely to yield a complete picture of what is needed to implement an evidence-based multi-level health intervention. Additionally, longitudinal assessment, both formal and informal, can facilitate knowledge of what adaptations will be needed to address key barriers related to the intervention itself (11). Finally, for patient-reported barriers, increased patient involvement may be needed to better identify the optimal implementation strategies to overcome these barriers.

One of the unique features of this study is the use of CFIR (23) to integrate implementation strategies used by the implementation team with qualitative evaluation of both the barriers targeted and perceptions of the strategies themselves. This differs from prior studies using LISTS to describe strategies (22) in the ability to provide additional nuance to how these strategies were perceived and valued by those engaging with the intervention via qualitative data. Furthermore, the use of direct feedback from participants to describe the application of these implementation strategies has the potential to help future implementers understand how to best categorize improvement efforts within the context of implementation science. Ultimately, provision of examples will be key to improving the collaboration between individuals in the health system working in quality improvement and practice transformation with the field of implementation science (34).

The need to explicitly study implementation of healthcare delivery interventions is perhaps more pressing in the contemporary landscape where health systems are required to rapidly implement healthcare delivery interventions, such as ePROs for symptom monitoring, within value-based healthcare payment reform models (35). In oncology, ePRO implementation was considered to be a barrier to participating in Medicare's payment reform, the EOM (1), resulting in only modest initial participation in EOM with 44 practices compared to the previous model, the Oncology Care Model, which included 190 practices (36). In June 2024, Medicare announced a second opportunity for practices to enter into EOM participation that included additional financial support for practices (37). Thus, the cohort of sites available to immediately benefit from guidance on RSM is substantial. Given the goal of implementation science “to support improvements in health and healthcare at scale” (38), the field is uniquely poised to play a critical role in the success of payment models that promote patient-centered care.

This study has several key limitations. First, the implementation was focused on two academic centers in the deep South, where cultural context may differ from other regions of the country and from private practice settings. However, we anticipate many of the challenges to be shared with other clinical settings. Also, given the available sample of interview participants at two sites, we did not have sufficient numbers to consider the perspectives of administrators or other senior-level leaders who may have different perspectives on barriers to implementation. The research team included some individuals participating in implementation, which could lead to bias. This was mitigated through extensive discussion with members of the multidisciplinary research team not directly involved in implementation until consensus was reached. This study aimed to provide a holistic overview of the implementation of RSM and did not delve into the nuances of how individual barriers and implementation strategies influenced specific, more vulnerable populations (e.g., the elderly, low literacy or digital health literacy, Black patients, rural residents), and further work will be needed to optimize implementation within the context of equitable care delivery. In addition, patients provided perspectives on their individual experiences, which may not fully capture changes made by the implementation team over time and thus patients may have been less aware of implementation strategies used to overcome barriers. It is also possible that patients who participated in the interviews had a more favorable view of RSM than those who declined, which may limit capture of some patient barriers. We were not able to conduct member checking, so some perspectives may have been missed. Finally, the study focused on the early implementation of ePROs in real-world settings rather than the entire trajectory of implementation including the maintenance phase, which is reflected in the focus on barriers at the level of the implementation setting and the implementer characteristics, as well as on implementation outcomes of feasibility and adoption. In contrast, few strategies focused on cost and sustainability, which are expected to be dominant for programs aiming for long-term maintenance. A deeper understanding of the phases of implementation remains a knowledge gap.

## Conclusion

Early implementation of RSM is complex, requiring multiple implementation strategies to overcome barriers at multiple CFIR (23) levels. Future work is needed to leverage learnings from rigorous implementation studies to facilitate the rapid adoption of RSM into the oncology community to support ongoing payment reform.

## Data Availability

Data from this analysis will be made upon reasonable request and appropriate approvals.
