# Supplementary material for: Barriers encountered and implementation strategies deployed to facilitate real-world remote symptom monitoring in patients receiving active cancer treatment
Source: Front Health Serv. 2026 Aug 12;6:1875180. doi: 10.3389/frhs.2026.1875180 (PMC13506834; doi:10.3389/frhs.2026.1875180)
Supplement: Supplementary file 1 [file Datasheet1.docx]

Supplemental Table 1: Barriers that remain unaddressed

| **Identified by:** | **CFIR Domains** | **Constructs (Determinants)** | **Specific Barrier** | **Barrier Quotes** |
| --- | --- | --- | --- | --- |
| Both Patient and Clinical Team Member | Intervention Characteristics | Complexity | **Survey Link Seen as Spam or Junk** | "and then one time it was actually in my spam box." MCI Patient 7  "I get a bunch of junk calls. So you might have been calling me and I might have been, if I didn't recognize, who it was, I could have been just sending that to voicemail that could have been what was going on." UAB Patient 2  "And make sure that when you call or text that it's not lumped in with junk." UAB Patient 2 |
|  | Intervention Characteristics | Design Quality and Packaging | **Patient Understanding of Symptom Severity** | "Now and they'll call for, I guess they would call for severe things, but sometimes they'll put stuff in, sometimes they are over exaggerating things and it's not as bad as they make it sound, and then you call them and they're like, "Oh yeah, that, no, I'm fine. That was just..."" Y1 MCI Provider 1  "Well, it's hard to say whether it's a mild symptom or a moderate symptom ... Sometimes I want to say, "Well, this past week I was normal, but I don't think I'm over this yet."" Y2 UAB Patient 19 |
|  | Intervention Characteristics | Design Quality and Packaging | **Patient Understanding of Hospitalization Question** | "A lot of patients are marking themselves hospitalized because they're in infusion and they think that that's them being hospitalized.  So [non-clinical navigator] and I have talked with [Carevive Support] about maybe getting something added to where it makes it clear to them that if you're inpatient and you're admitted, that's what classifies hospitalized, because this has happened several times now and I have to go mark them unhospitalized, because I'm like, "No, you're just sitting down in infusion."" MCI Provider 13 |
|  | Inner Setting | Networks and Communications | **Physician not Using Dashboard** | "But I think if the provider would mention it in the visit, "Oh, I've noticed you've been feeling nauseous or have a lot of fatigue over the last few weeks through your surveys." And they're like, "Oh yeah, I should probably continue to do those surveys cause people are looking at them."" Y1 MCI Provider 7  "I already have my own review of systems with patients, so I don't need to [use the dashboard] unless there was something that was triggered and was escalated.." Y2 UAB Provider 15  "So, they ask me the same questions that are already on the survey, and maybe I should have said, "Well, I filled out my survey. Did you not look at it?" They just gather the same information orally at each visit that I have already put on this." Y2 UAB Patient 12 |
| Patient Only | Delivery Setting Processes | Engaging | **Caregiver Said No on Initial Approach** | "I didn't. I was not asked. My husband made that decision for me. I'd be happy to do that." UAB Patient 10 |
|  | Delivery Setting Processes | Engaging | **Confidentiality and Data Access Concerns** | "Just wanted to know [who the data is shared with] because the information that you may be asking may be kind of sensitive, may be too personal." UAB Patient 3  "Some of them may be reluctant to talk to you, because they're not really talking to the doctor, and they think they just telling stuff to anybody." UAB Patient 11 |
|  | Delivery Setting Processes | Engaging | **Previous Medical Experiences Result in Symptom Intervention Mistrust** | "When I kind of talked to him about it [symptom burden], it was like a brushoff when I first told him about it." MCI Patient 7  "I did and they really didn't do anything [address symptoms]." UAB Patient 14 |
|  | Intervention Characteristics | Design Quality and Packaging | **Overly Basic and Repetitive Symptom Management Info Received** | "I guess, because basically most of the time when I filled it out, it was about the same" UAB Patient 9  "No, that it's just, well, I feel like it's redundant, and I can see how some people might need that, but I don't need it because I remember it." UAB Patient 19 |
|  | Intervention Characteristics | Relative Advantage | **Able to Self-Manage Symptoms** | "I guess I just figured out how to manage it myself and just hadn't really looked at that part." MCI Patient 6  "I knew how to handle them, and so I didn't feel like I needed extra help or anything like that." MCI Patient 20 |
|  | Intervention Characteristics | Relative Advantage | **Experiences No, Mild, Expected or Inactionable Symptoms** | "Like I said, I don't have that very many symptoms so... Although I did read it. I have mild symptoms. Like I said, my worst thing is those fatigue problems." MCI Patient 1  "Yeah. She sends me a thing every Tuesday. Hi, it's Clara. And I answer the same questions, I'm the same way, usually. I'm just not having a problem." UAB Patient 16 |
| Provider Only | Delivery Setting Processes | Engaging | **Concern that Caregiver Completed Surveys are Inaccurate** | "I don't put a lot of my elderly patients on there, and I think that's because I'm fearful that they're not going to answer, or their family's going to answer for them, and I want to hear from them versus their family. " UAB Provider 3  "I'm not aware of an issue. I do know of one of my patients whose spouse reported that they were deceased through this just to let the clinic know, but that was just a one-time notification." Y2 UAB Provider 16 |
|  | Delivery Setting Processes | Engaging | **Difficulty Identifying Elligible Patients** | "I mean, I think before we [fully rolled-out], it wasn't necessarily a challenge, but it did take a lot of time to screen everyone's clinic schedule to find eligible patients." MCI, Provider 7 |
|  | Delivery Setting Processes | Executing | **Limited Staffing and Staff Retention Problems** | "So especially with our rollout schedule, sometimes we have had to push dates back only because we've had nurses to leave, we had doctors to leave, and then sometimes staffing purposes as well. We didn't have enough people to actually enroll those people into the surveys to receive the surveys." UAB Provider 4  "We're a very small manpower. Me and our two other team members approach all of the patients at our site. So it is a lot." MCI Provider 13 |
|  | Intervention Characteristics | Adaptability | **Need for Regimen Specific Surveys** | "On the one hand for data collection, ease of general survey for symptoms. On the other hand having specific symptoms for say targeted therapy or immunotherapy as opposed to traditional chemotherapy. Or having those rare side effects of a particular drug would be helpful." UAB, Provider 6  "You could probably tailor these to either disease groups or to regimens if someone ever wanted to spend the time to take those however many items and carve them down. Because I don't have to worry about hot flashes. That's just not a thing. It doesn't come up much. Or at least it's unrelated to my chemo, whereas other things that's really important. But overall, I think it's fine." UAB Provider 10 |
|  | Intervention Characteristics | Adaptability | **Need for Survey Continuation and Modification after Treatment End** | "But if someone has completed chemo, for example, it is important to [continue to track symptoms]. I think from my end, for those up to four to six months after, how are they feeling in terms of neuropathy, for example? Is that neuropathy getting better? Is it more persistent? I don't anticipate of course any of the other shorter-term side effects, like nausea or any GI issues, but capturing those sort of more permanent or long-term side effects." UAB, Provider 6 |
|  | Intervention Characteristics | Complexity | **Not Available for All Patients** | "And if it's something you do in every patient, you know it's coming, you know it's there. And I think you, one, you remember to do it more often, you don't forget to address those areas." MCI Provider 8 |
|  | Intervention Characteristics | Complexity | **Patient with Multiple Diagnoses** | "I know the other day I ran into an issue where we had a patient who has two separate diagnoses, and so she currently has, or she has previously had breast cancer, and she's on maintenance treatment for that. But she currently has a vaginal cancer, and when we were enrolling her, she was a new patient starting treatment, and when we were enrolling her, it automatically assigned her as a breast patient." UAB, Provider 6 |
|  | Intervention Characteristics | Cost | **Needs More Funding and Support** | "I think it needs more support. It needs more financial support from institutions. It needs a bigger team." UAB, Provider 5 |
|  | Intervention Characteristics | Design Quality and Packaging | **Dashboard Difficult to Navigate** | "I think, however, the interface can be slightly more cluttered and it does depend on... It's very rich, options, but it's not easy to navigate. And it almost always needs somebody to tell you, "You have to click this. You have to do that." The interface, making it more seamless, more user-friendly, I think, will help increase its usage." UAB, Provider 5 |
|  | Intervention Characteristics | Design Quality and Packaging | **Need Ability to Document Compliance Calls** | "I wish that there was an option in Carevive to log a call. If you call a patient about compliance, first week ... I wish there was a way to log that. We have made our own log in Excel, but I wish there was a way within Carevive to document that, because I feel like that's useful to know this person's never filling out their surveys, but they're also never answering the phone." MCI, Provider 6 |
|  | Intervention Characteristics | Design Quality and Packaging | **Need Ability to Filter Alerts by Severity (Prioritization)** | "Probably thinking of ways to minimize some of the low acuity. Or give them the ability to filter, for example, by severity, so that their inbox is not cluttered with a lot of... Yeah." UAB, Provider 5  "And almost sometimes I wish that there was an exclamation point on some of them that I needed to go to ASAP." MCI Providers 11 and 12 |
|  | Intervention Characteristics | Design Quality and Packaging | **Not All Symptoms Present** | "I mean, depression, I think we still need to hear that, and that's actionable as well." MCI, Provider 9  "It depends on the disease and the treatment type, right? So there's never going to be a universal set of symptoms. A patient on immunotherapy, the diarrhea may be critically important. A patient getting 5-FU... I don't know if you've practiced breast oncology. Or maybe, I don't know, a cardiac symptom might be important for someone getting anthracyclines. So I don't think there will be a universally agreed upon set of symptoms that will be perfect." UAB Provider 15 |
|  | Intervention Characteristics | Trialability | **Cancer Type Specific Roll Out** | "The hesitation I have a little bit by disease group is that, especially at a small institution, not everyone does just one disease type. And you have some that are just general medical oncologists, where [they] see everything." MCI Provider 8 |
|  | Inner Setting | Networks and Communications | **Hard to Contact Patients by Phone** | "We've had a lot of trouble with people answering the phone, and I don't know if people are tired of everything being by phone, or whatever the case may be that they're just over any kind of over the phone communication." UAB, Provider 6 |
|  | Inner Setting | Networks and Communications | **Need Ability to Text/Instant Message Patients** | "And one patient was just like, "well, I wish they could just text me back." And I was like, well, they can't text back, they could call you." UAB, Provider 4 |
|  | Inner Setting | Structural Characteristics | **Better Integration with EMR Needed** | "The integration is somewhat partial or less than ideal. It would be much better if the integration was ordered directly in real time, directly inside the medical record, not a link within the medical record that opens a different website, a different platform, right?" UAB Provider 15 |

**Supplemental Table 2**: Implementation strategy, category of implementation strategy, primary barrier targeted, primary implementation outcome targeted during initial implementation and scale-up of ePRO based remote symptom monitoring.

| **Strategy Category** | **Strategy** | **Barrier** | **Barrier details** | **Primary Outcome Targeted** |
| --- | --- | --- | --- | --- |
| **Use Evaluative and Iterative Strategies** | **Assess for readiness and identify barriers and facilitators** | Characteristics of the individuals implementing and supporting the intervention | Knowledge and Beliefs about the Intervention; Self efficacy; Individual stage of change; Individual Identification with Organization | Feasibility |
|  |  | Process in the delivery setting | Planning |  |
|  | **Audit and provide feedback** | Characteristics of individuals implementing and supporting the intervention | Individual stage of change | Fidelity |
|  |  | Process in the delivery setting | Reflecting and Evaluating |  |
|  | **Develop and implement tools for quality monitoring** | Inner setting | Implementation climate | Feasibility |
|  |  |  | Readiness for implementation |  |
|  |  | Process in the delivery setting | Reflecting and Evaluating | Fidelity |
|  | **Obtain and use patients/consumers and family feedback** | Process in the delivery setting | Engaging; Executing | Reach |
|  | **Purposefully reexamine the implementation** | Process in the delivery setting | Planning; Executing; Reflecting and Evaluating | Sustainability |
| **Provide interactive assistance** | **Centralize technical assistance** | Characteristics of individuals implementing and supporting the intervention | Design Quality and Packaging | Fidelity |
|  |  | Process in the delivery setting | Executing |  |
|  | **Facilitation**** | Characteristics of the intervention | Adaptability; Complexity; Design Quality and Packaging | Feasibility |
|  |  | Characteristics of the individuals implementing and supporting the intervention | Knowledge and Beliefs about the Intervention |  |
|  |  | Delivery Setting | Planning; Engaging; Reflecting and Evaluating |  |
|  |  | Characteristics of the individuals implementing and supporting the intervention | Self-efficacy; Individual Stage of Change | Adoption |
|  |  |  | Self-efficacy; Individual Stage of Change | Reach |
|  | **Provide local technical assistance** | Barriers related to the characteristics of the intervention | Complexity; Design Quality and Packaging | Fidelity |
|  |  | Delivery Setting | Executing |  |
| **Adapt and Tailor to the Context** | **Promote adaptability of the EBP** | Characteristics of individuals implementing and supporting the intervention | Self -efficacy | Acceptability |
|  |  | Process in the delivery setting | Executing |  |
|  | **Tailor strategies** | Characteristics of the intervention | Adaptability; Complexity; Design Quality Packaging | Adoption |
|  |  | Outer Setting | Patient needs and Resources; Peer Pressure |  |
|  |  | Inner setting | Implementation climate; Readiness for Implementation |  |
|  |  | Characteristics of the individuals implementing and supporting the intervention | Knowledge and Beliefs about the Intervention; Self efficacy; Individual stage of change |  |
|  |  | Process in the delivery setting | Engaging; Executing |  |
|  | **Use data warehousing techniques** | Process in the delivery setting | Reflecting and Evaluating | Sustainability |
| **Develop Stakeholder Interrelationships** | **Capture and share local knowledge** | Characteristics of individuals implementing and supporting the intervention | Adaptability; Trialability; Complexity; Design Quality and Packaging; Cost | Acceptability |
|  |  | Inner setting | Networks and Communication; Implementation Climate |  |
|  |  | Process in the delivery setting | Engaging; Executing; Reflecting and Evaluating |  |
|  | **Involve executive boards** | Inner setting | Readiness for implementation | Feasibility |
|  | **Obtain formal commitments** | Process in the delivery setting | Planning; Engaging |  |
|  |  | Characteristics of the individuals implementing and supporting the intervention | Knowledge and Beliefs about the Intervention; Self-efficacy; Individual Stage of Change |  |
|  | **Promote network weaving** | Characteristics of the individuals implementing and supporting the intervention | Knowledge and Beliefs about the Intervention; Self efficacy; Individual stage of change | Adoption |
|  | **Use advisory boards and workgroups** | Outer Setting | Cosmopolitanism; Peer Pressure; External Policy and Incentives | Sustainability |
|  |  | Inner setting | Implementation climate; Readiness for Implementation |  |
|  | **Develop academic partnerships** | Characteristics of the intervention | Evidence Strength and Quality; Complexity; Design Quality and Packaging | Fidelity |
|  | **Inform local opinion leaders** | Characteristics of the individuals implementing and supporting the intervention | Knowledge and Beliefs about the Intervention; Self efficacy; Individual stage of change | Adoption |
|  | **Identify and prepare champions** | Characteristics of the individuals implementing and supporting the intervention | Knowledge and Beliefs about the Intervention; Self efficacy; Individual stage of change | Adoption |
|  | **Use an implementation advisor** | Characteristics of individuals implementing and supporting the intervention | Adaptability; Trialability | Feasibility |
|  |  | Process in the delivery setting | Planning; Executing |  |
| **Train and Educate Stakeholders** | **Conduct educational meetings** | Delivery Setting | Engaging; Reflecting and Evaluating | Acceptability |
|  | **Conduct educational outreach visits**** | Characteristics of the individuals implementing and supporting the intervention | Knowledge and Beliefs about the Intervention; Self efficacy; Individual stage of change | Adoption |
|  |  | Characteristics of the individuals implementing and supporting the intervention | Knowledge and Beliefs about the Intervention; Self efficacy; Individual stage of change | Adoption |
|  | **Develop educational materials**** | Characteristics of the intervention | Adaptability; Complexity; Design Quality Packaging; Costs | Feasibility |
|  |  | Characteristics of the individuals implementing and supporting the intervention | Self-efficacy; Individual Stage of Change |  |
|  |  | Process in the delivery setting | Executing |  |
|  | **Conduct ongoing training** | Process in the delivery setting | Engaging; Champions | Sustainability |
|  | **Create a learning collaborative** | Characteristics of the intervention | Adaptability; Trialability; Complexity; Design Quality and Packaging; Cost | Feasibility |
|  |  | Inner setting | Networks and communications; Culture; Implementation Climate; Readiness for Implementation |  |
|  |  | Characteristics of the individuals implementing and supporting the intervention | Knowledge and Beliefs about the Intervention; Self efficacy; Individual Identification with Organization |  |
|  |  | Process in the delivery setting | Planning; Reflecting and Evaluating |  |
|  | **Distribute educational materials**** | Characteristics of the intervention | Complexity; Design Quality and Packaging | Feasibility |
|  |  | Characteristics of the individuals implementing and supporting the intervention | Self-efficacy; Individual Stage of Change |  |
|  |  | Characteristics of the intervention | Complexity; Design Quality and Packaging | Reach |
|  | **Make training dynamic** | Characteristics of the individuals implementing and supporting the intervention | Self-efficacy; Individual Stage of Change | Adoption |
|  | **Provide ongoing consultation** | Characteristics of the individuals implementing and supporting the intervention | Self-efficacy; Individual Stage of Change | Adoption |
| **Support Clinicians** | **Develop resource sharing agreements**** | Characteristics of the intervention | Adaptability; Trialability; Complexity; Design Quality and Packaging; Cost | Feasibility |
|  |  | Inner setting | Structural Characteristics |  |
|  |  | Delivery Setting | Planning; Executing |  |
|  | **Facilitate relay of clinical data to providers** | Characteristics of the individuals implementing and supporting the intervention | Other Personal Attributes | Feasibility |
|  |  | Process in the delivery setting | Executing |  |
|  | **Remind clinicians** | Characteristics of the individuals implementing and supporting the intervention | Other Personal Attributes | Feasibility |
|  |  | Process in the delivery setting | Executing |  |
|  | **Revise professional roles** | Process in the delivery setting | Planning; Executing | Feasibility |
|  |  |  |  |  |
| **Engage Consumers** | **Intervene with patients/consumers to enhance uptake and adherence** | Characteristics of the intervention | Design Quality and Packaging | Acceptability |
|  |  | Process in the delivery setting | Reflecting and Evaluating |  |
|  |  | Process in the delivery setting | Executing | Fidelity |
|  | **Involve patients/consumers and family members**** | Outer Setting | Patient needs and Resources | Appropriateness |
|  |  | Outer Setting | Patient needs and Resources | Acceptability |
|  | **Prepare patients/consumers to be active participants** | Characteristics of the intervention | Evidence Strength and Quality; Complexity; Design Quality and Packaging | Fidelity |
| **Utilize Financial Strategies** | **Access new funding**** | Inner setting | Readiness for implementation | Reach |
|  |  |  |  |  |
|  |  | Characteristics of the intervention | Design Quality and Packaging | Reach |
| **Change Infrastructure** | **Change physical structure and equipment** | Characteristics of the intervention | Design Quality and Packaging | Feasibility |
|  |  | Inner setting | Networks and Communication | Adoption |
|  | **Change record systems** | Process in the delivery setting | Engaging; Executing; Reflecting & Evaluating | Fidelity |
|  |  | Characteristics of the intervention | Adaptability; Design Quality and Packaging; Cost | Acceptability |
|  |  | Characteristics of the individuals implementing and supporting the intervention | Individual stage of change |  |
|  |  | Process in the delivery setting | Executing |  |
|  | **Change service sites** | Process in the delivery setting | Engaging | Acceptability |
|  | **Mandate change** | Characteristics of the individuals implementing and supporting the intervention | Individual stage of change | Adoption |
